# *Phytophthora capsici* carries and differentially expresses genes for the RNA interference pathway

**DOI:** 10.1371/journal.pone.0333769

**Published:** 2026-01-30

**Authors:** Jacobo Sevillano-Serrano, Fernando Uriel Rojas-Rojas, Alfonso Méndez-Bravo, Nancy Calderón-Cortés, Harumi Shimada-Beltrán, Julio Vega-Arreguín

**Affiliations:** 1 Laboratorio de Ciencias Agrogenómicas and Laboratorio Nacional PlanTECC. Escuela Nacional de Estudios Superiores, Unidad León-UNAM, Blvd. UNAM, León, Gto, México; 2 Posgrado en Ciencias Biológicas, Universidad Nacional Autónoma de México. Unidad de Posgrado, Edificio D, 1° Piso, Circuito de Posgrados, Ciudad Universitaria, Coyoacán, Ciudad de México, México; 3 Escuela Nacional de Estudios Superiores Unidad Morelia-UNAM. Antigua Carretera a Pátzcuaro, Col. Ex Hacienda de San José de la Huerta, Morelia Mich, México; 4 SECIHTI, Av. Insurgentes Sur, Colonia Crédito Constructor, Demarcación Territorial Benito Juárez, Ciudad de México, México; Benemérita Universidad Autónoma de Puebla: Benemerita Universidad Autonoma de Puebla, MEXICO

## Abstract

The RNA interference (RNAi) pathway is an epigenetic mechanism that has recently gained attention for its role in regulating the virulence of plant pathogens. However, little is known about this gene silencing pathway in *Phytophthora capsici*, a broad-host-range pathogen that affects many important food crops. In the present study, we identified key genes and proteins involved in the synthesis, transport, and processing of sRNAs using *in silico* approaches based on the reference genome and proteome, and through transcriptional analysis of *P. capsici*. Our results showed that the *P. capsici* genome encodes Dcl1, Dcl2, Exportin-5, Rdr, and six Ago proteins, suggesting the presence of a complete RNAi pathway in this pathogen. These genes were syntenic and phylogenetically related to those of other oomycetes in the genus *Phytophthora*. We also analyzed their expression levels after infecting chili pepper and broccoli across two generations, revealing different expression patterns depending on the infection history of the pathogen. To our knowledge, this is the first report on the *EXPORTIN-5* gene in *P. capsici* and other oomycetes. Additionally, the expression of all these RNAi-related genes in the pathogen after isolation from different hosts suggests that the host may influence the RNAi pathway of *P. capsici*. This study paves the way for functional studies to confirm the role of RNAi in regulating virulence in *P. capsici*.

## Introduction

*Phytophthora capsici* is a destructive oomycete pathogen [1,2] belonging to the Stramenopila kingdom that threatens agricultural and natural ecosystems [3–5]. Although oomycetes resemble filamentous fungi, they are phylogenetically related to aquatic organisms such as diatoms and algae [6,7]. *P. capsici* can infect a wide range of hosts in laboratory, greenhouse, and field conditions, affecting at least 26 plant families, including both ornamental and native plants. This pathogen poses a significant threat to food security [2,8] because it causes diseases in various crops worldwide, including chili peppers, cucumbers, watermelons, and squash [9–11]. *P. capsici* is a hemibiotrophic, soil-borne pathogen that exhibits mixed reproduction [12,13] and can infect host plants at any growth stage, leading to seedling death, crown rot, leaf blight, and fruit rot [14]. These characteristics make it an excellent model organism for studying how plant pathogens adapt to diverse hosts, including the epigenetic mechanisms of gene silencing that may regulate this process [13].

Gene silencing mediated by small RNAs (sRNAs) occurs in oomycetes, including *P. infestans*, *P. sojae,* and *P. parasitica* [15–18]*,* but the molecular mechanisms underlying this process are not well characterized in these organisms [19]. Small RNAs are short RNA sequences, 20–40 nucleotides (nt) long, that do not encode proteins but play important roles in various eukaryotic pathogens, including the regulation of endogenous biological processes and host interactions [20–25]. Generally, small RNAs involved in the RNA interference pathway are small interfering RNAs (siRNAs) and microRNAs (miRNAs) [26]. In plants [27] and oomycetes, these sRNAs are synthesized and processed by Dicer-like enzymes (Dcl), which are ribonuclease enzymes that cut sRNAs into different sizes [19]. In contrast, in animals and mammals, this process occurs via Drosha and Dicer enzymes [28,29]. sRNAs are transported from the nucleus to the cytoplasm via Exportin-5 (mammals)/Hasty [30,31], an enzyme found in the nuclear membrane [32]. In the cytoplasm, they bind to an Argonaute (Ago) family enzyme to form the RNA-induced silencing complex (RISC). This complex binds to a target messenger RNA (mRNA) through base complementarity between the siRNA and mRNA [33]. The Ago enzyme then performs its ribonuclease activity, cleaving the target mRNA. This leads to the regulation of the target mRNA at the post-transcriptional level, a process known as post-transcriptional gene silencing (PTGS). Later, the RNA-dependent RNA polymerase (Rdr) may recognize the cleaved mRNA fragments and synthesize double-stranded RNAs (dsRNAs). These can be incorporated into the Dcl enzyme, generating small secondary RNAs that amplify the silencing signal for a specific gene by binding to Ago enzymes and recognizing complementary sequences [34,35]. In *Phytophthora* species, little is known regarding how gene silencing is mediated by sRNAs, the enzymes involved, and the regulation of pathogenicity [36]. Indeed, the few reports on epigenetic regulation in pathogenic oomycetes have mainly focused on histone methylation, acetylation, and deacetylation, long non-coding RNAs [37], and small RNAs related to effector genes [38–41]. Recently, *P. capsici* was found to express small RNAs during different life stages [40], but the roles of the genes involved in the RNAi pathway remain unknown.

In this study, we aimed to identify, *in silico* characterize, and evaluate the expression of key genes encoding enzymes involved in the RNA interference pathway in *P. capsici,* and to determine their expression patterns in the pathogen after infecting different host plants. This analysis could support future functional studies to describe the specific roles of the RNAi pathway in *P. capsici* pathogenicity and growth.

## Materials and methods

### Identification and phylogenetic analysis of sRNA-related enzymes in *P. capsici*

Dcl, Exportin-5 (Exp5), Ago, and Rdr protein sequences were searched in the *P. capsici* LT1534 proteome (GCA_000325885.1) from the PhycoCosm portal of the Joint Genome Institute (JGI) [13] using Hidden Markov Models (HMM) based on the seed alignments of functional domains of each enzyme family obtained from the Pfam v35.0 database [42].

The HMM probability distributions for the characteristic domains of each enzyme were generated using HMMER v3.3.2 [43], and proteins containing the specified number and position of characteristic domains within each protein family were identified. Proteins with two RNAase III domains at the C-terminus were classified as Dcl enzymes. Sequences containing PAZ, MID, and PIWI domains were classified as Ago proteins. To be considered Rdr, sequences must contain an RdRP domain. Sequences lacking a domain were considered pseudogenes/proteins.

The conserved domains Xpo1 and exportin-5 from *Xenopus tropicalis* [44] Exportin-5 sequence were used to build an HMM with HMMER c3.3.2 [42,43] to identify the Exp5 protein in *P. capsici.* All gene models were verified and manually curated to validate our findings. Additionally, transcript evidence was confirmed by RNA-seq data from our group [45] (S1 Table).

For phylogenetic analysis, homologous protein sequences from other oomycetes were retrieved from the NCBI database. Homologous protein sequences of *Arabidopsis thaliana*, *H. sapiens*, and *Mus musculus* served as outgroups. Subsequently, multiple sequence alignments for each protein family were performed using MUSCLE in MEGA11 [46], followed by sequence curation with GBlocks v0.91b [47]. Phylogenetic reconstruction was then performed using maximum likelihood (ML) with the Akaike Information Criterion (AIC) in PhyML [48], selecting the best-fitting substitution model, and supporting branches with 100 bootstrap iterations. Tree topology visualization was performed using TreeDyn v1.98.3 and Evolview [49,50].

Characteristic domains were identified using the NCBI Conserved Domain Search tool [51] and visualized with TBtools-II v1.120 [52]. The Ago nomenclature reported for other oomycetes has been retained [53]. However, consecutive numbers were assigned to distinguish proteins that had not been detected in different pathogens. Lastly, the subcellular localization of the genes was predicted using Predict Protein LocTree3v4.0 [54].

### Characteristics of Exportin-5 of *P. capsici*

Since no genes or proteins involved in the nuclear export of sRNA have been identified in *Phytophthora* species, we analyzed the genes and proteins in *P. capsici* that exhibit sRNA export features similar to those of the *EXPORTIN-5* genes reported in other organisms. The genetic neighborhood of the *P. capsici* EXP5 gene was examined using the genome browser tool at the Joint Genome Institute (JGI) [55] and compared with *P. infestans*, *P. sojae*, and *P. ramorum*. The features of *P. capsici* scaffolds were visualized using the synteny tool on the JGI website [55]. To identify the conserved domains IBN_N, Xpo1, and Exportin-5, multiple sequence alignments of Exp5 proteins from *Phytophthora*, *H. sapiens*, and *M. musculus* were performed using EMBOSS Needle [56] and MUSCLE [46]. The abundance of amino acids at each position in the primary sequence of these proteins was analyzed using the WebLogo program [57].

### Synteny analysis and gene interaction networks

To understand the conservation of genes encoding key enzymes of the RNAi pathway in *P. capsici*, we conducted a synteny and collinearity analysis using the Synteny OneStep MCScanX tool [58] in TBtools-II v1.120 [52], across the genomes of *P. capsici* [59], *P. infestans* [60], *P. sojae* [61], and *P. ramorum* [61], obtained from the NCBI and JGI databases.

To predict whether the identified proteins in *P. capsici* could interact with each other or with related proteins, we performed *in silico* protein-protein interaction analysis. Protein-protein interaction networks for the Dcl, Exp5, Ago, and Rdr proteins were built in the STRING v11.5 database [62], with a medium confidence interaction score (0.40), and limiting the 1st and 2nd shell to no more than five interactions. The chosen confidence score indicates a medium level of confidence in the predicted protein-protein interaction or its biological significance. The 1st and 2nd shell parameters “no more than five interactions” specify that only the top five most probable interactions between the analyzed proteins and the top five related proteins are displayed.

### Influence of hosts on the expression of *P. capsici* RNAi pathway-related genes

#### Infection and isolation of *P. capsici* from plant tissue.

To determine the expression patterns of RNAi pathway-related genes in *P. capsici* after infecting different hosts, leaves from chili and broccoli were collected from plants grown in greenhouse conditions for 4–6 weeks. These leaves were infected with agar plugs containing fresh mycelium of *P. capsici* D3 as described previously [63]. Infection assays were performed as follows: detached leaves were washed with 70% ethanol, rinsed three times with sterile water, and then placed in a sterile humidity chamber with the underside facing up. Next, a ~ 2 mm wound was made in the center of each leaf and inoculated with a 5 mm diameter plug of 8-day-old *P. capsici* D3 mycelium. The leaves were incubated at 28°C in the dark, and infection was monitored at 24, 48, and 72 hours post-inoculation (hpi). Each experiment was performed six times using four leaves per plant species. The infected area on each leaf was measured using ImageJ software [64], and statistical analysis, including normality tests and ANOVA (p < 0.05), was performed.

*P. capsici* D3 was isolated from infected tissues of chili and broccoli (primary infections, I-chili and I-broccoli) at 72 hpi and grown in the dark at 28°C on Petri dishes with V8 medium [65] supplemented with antimicrobials including benomyl (100 mg/L), rifampicin (80 mg/L), ampicillin (100 mg/L), streptomycin (50 mg/L), and kanamycin (50 mg/L). These first isolates of *P. capsici* D3 (Pc-chili and Pc-broccoli) were used to re-infect new chili plant tissues (secondary infections, I-ch-ch and I-br-ch). The isolates from these secondary infections (Pc-ch-ch and Pc-br-ch) were cultivated similarly to those from the primary infections. Both primary and secondary isolates of *P. capsici* D3, obtained after infecting the two hosts, were grown for 8 days, and the mycelia were collected for total RNA extraction, which was then used in gene expression assays.

#### RT-qPCR assays.

Relative gene expression in the mycelia of the parental *P. capsici* D3 without infection history (control), primary isolates (Pc-chili, Pc-broccoli), and secondary isolates (Pc-ch-ch, Pc-br-ch) was quantified by RT-qPCR. Primers designed and used for each gene are listed in S3 Table.

To determine the expression of *DCL*, *EXP5*, *AGO*, and *RDR* genes in *P. capsici,* primers were designed using the NCBI Primer–BLAST program [66] and validated by end-point PCR with a temperature gradient, using *P. capsici* D3 genomic DNA obtained with the Plant/seed DNA Miniprep kit (ZYMO RESEARCH) following the manufacturer’s instructions.

Gene expression was analyzed using quantitative PCR (qPCR) with cDNA libraries prepared by reverse transcription (RT) from total RNA from five *P. capsici* mycelial samples (D3, Pc-chili, Pc-broccoli, Pc-ch-ch, and Pc-br-ch) obtained as described above. Total RNA was extracted using the Quick-RNA Plant Miniprep kit (Zymo Research) according to the manufacturer’s guidelines, and its integrity was verified by agarose gel electrophoresis and NanoDrop. cDNA synthesis was performed using the Revert First Strand cDNA Synthesis kit (Thermo Scientific) through reverse transcription reactions of 20 µL containing 4 µL 5X Buffer, 2 µL dNTPs (10 mM), 0.5 µL RiboLock, 1 µL oligo(dT)_18_, 10 units of RevertAid Reverse Transcriptase, 1000 ng of total RNA, and nuclease-free water in a thermal cycler with the following program: 42 °C for 60 min, 70 °C for 5 min, and hold at 4 °C.

qPCR was performed in 10 µL reactions consisting of 5 µL SYBR Green Real-Time PCR Master Mix 2X (Applied Biosystems | Thermo Fisher Scientific), 1 µL Forward Primer (2 mM), 1 µL Reverse Primer (2 mM), 1 µL cDNA (50 ng/µL), and 2 µL nuclease-free water on the StepOne Real-Time PCR System (Applied Biosystems). The qPCR conditions included an initial denaturation at 95°C for 10 min, followed by 40 cycles of 95°C for 15 s, then 54−68 °C for 30 s, and 72°C for 30 s; a third step involved increasing the temperature from 65°C by 0.3 °C every 0.1 min up to 95°C, and a final step at 95°C for 15 s. The 2ΔΔCt method [67] was used to determine the relative expression levels of each gene, with elongation factor-1α as the endogenous control gene. The assay was conducted in triplicate with two biological replicates. Graphs displaying gene expression levels, validated by ANOVA statistical analysis (p < 0.05), were generated using GraphPad Prism7 [68].

## Results

### *P. capsici* encodes *DCLs*, *EXP5*, *AGO*, and *RDR* genes that are phylogenetically related to those in other pathogenic oomycetes

After constructing HMMs and analyzing the *P. capsici* proteome, we identified key proteins related to sRNA biogenesis and processing*.* The characteristics of the genes encoding two *DCL* (*DCL1* and *DCL2*), one EXPORTIN-5 (*EXP5*), six Argonautes (1*, 2, 3, 4, 5*, and *6*), and one *RDR* are summarized in Table 1.

**Table 1:** Characteristics of genes encoding key enzymes of the *P. capsici* RNAi pathway.

Gene ID	Phyca11 ID	GO* ID	KOG/IPR** ID	KOG/IR*** Function	Exons	Locus (Phyca11)	Predicted location****
***DCL1***	511433	GO:0005634	KOG0701	RNA processing and modification	1	PHYCAscaffold_85:46098–48577 (+)	Nucleus
***DCL2***	18102	GO: 0005739	IPR002677	RNA binding, ribonuclease III	6	PHYCAscaffold_33:251889–255536 (-)	Mitochondria
***EXP5***	529988	GO:0005634	KOG2020	Intracellular trafficking, secretion and vesicular transport	2	PHYCAscaffold_52:377206–380861 (+)	Nucleus
***AGO1***	558569	GO:0005737	IPR12337	Nucleic acid binding, Polynucleotidyl transferase, Ribonuclease H	1	PHYCAscaffold_2:159343–161780 (-)	Cytoplasm
***AGO2***	64465	GO:0003676	IPR12337	Nucleic acid binding, Polynucleotidyl transferase, Ribonuclease H	1	PHYCAscaffold_19:453325–455820 (+)	Cytoplasm
***AGO3***	534306	GO:0003676	IPR12337	Nucleic acid binding, Polynucleotidyl transferase, Ribonuclease H	5	PHYCAscaffold_22:528439–531907 (-)	Cytoplasm
***AGO4***	54500	GO:0003676	IPR12337	Nucleic acid binding, Polynucleotidyl transferase, Ribonuclease H	1	PHYCAscaffold_22:528597–530987 (-)	Cytoplasm
***AGO5***	545808	GO:0003676	IPR12337	Nucleic acid binding, Polynucleotidyl transferase, Ribonuclease H	1	PHYCAscaffold_19:306374–310085 (+)	Cytoplasm
***AGO6***	106132	GO:0003676	IPR12337	Nucleic acid binding, Polynucleotidyl transferase, Ribonuclease H	1	PHYCAscaffold_12:883921–886497 (+)	Cytoplasm
***RDR***	107928	GO:0005634	KOG0988	RNA processing and modification	1	PHYCAscaffold_14:483193–484770 (+)	Cytoplasm

*GO ID: Identification data of the Gene Ontology database. **KOG/IPR ID: Identification data in Eukaryotic Orthologous Gene (KOG) and IPR: InterPro database. ***KOG/IR Function: Function description of Eukaryotic Orthologous Gene (KOG) and IR. Reference database: *Phytophthora capsici* LT1534 v11.0 (JGI).

**Fig 1:**
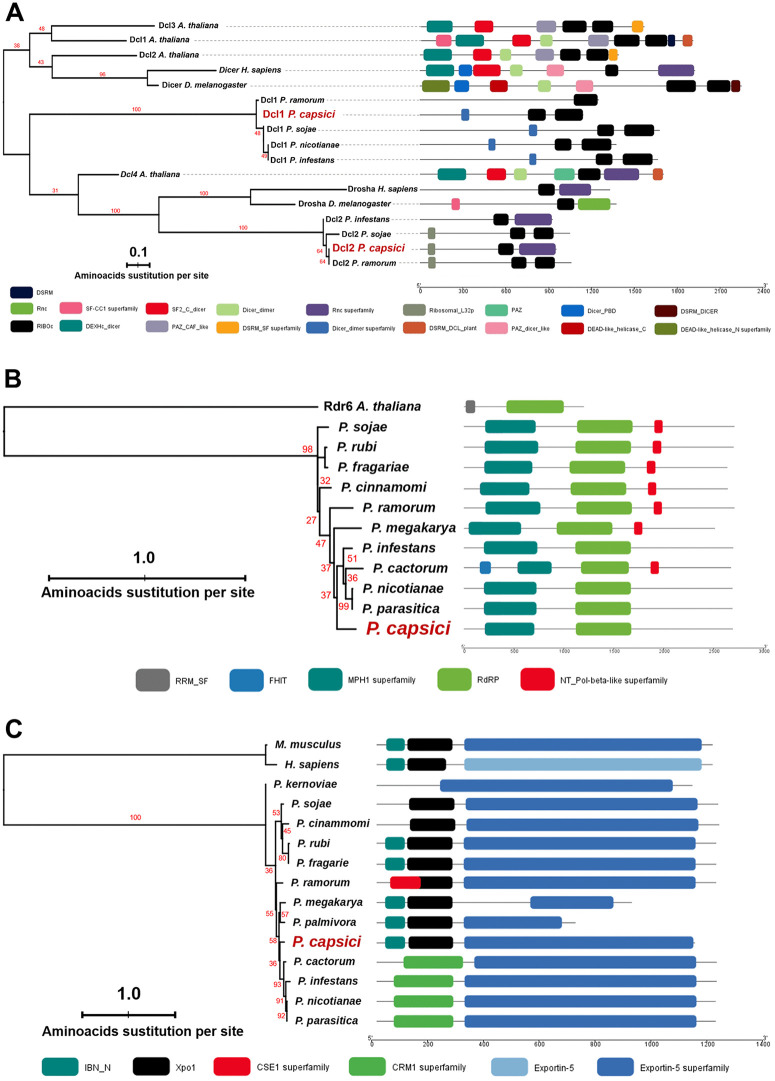
Phylogenetic reconstruction of Dcl, Rdr, and Exportin-5 in *Phytophthora* species. **(A)** Phylogenetic tree of Dicer-like proteins from *P. capsici* and other species. *A. thaliana* Dcl proteins 1-4, and Dicer and Drosha from *H. sapiens* and *M. musculus* were used as outgroups. **(B)** Phylogenetic tree of RNA-dependent RNA polymerase of *P. capsici* with Rdr from other oomycetes, using *A. thaliana* Rdr6 protein as an outgroup. **(C)** Phylogenetic tree of Exportin-5 proteins in *Phytophthora* species, with Exportin-5 from *M. musculus* and *H. sapiens* used as outgroups. The conserved domains of the proteins are highlighted in color on the right side of each panel. The scale bar indicates the size of the protein in amino acids. Colored rectangles with rounded corners indicate the protein domains.

Phylogenetic analysis of the amino acid sequences of Dcl1 and Dcl2 revealed that both *P. capsici* proteins are closely related to enzymes from *P. ramorum and P. sojae* (Fig 1A). Dcl1 was grouped into a clade with Dcl1 proteins from *P. infestans*, *P. sojae*, *P. nicotianae,* and *P. ramorum*. Meanwhile, Dcl2 was grouped with Dcl2 proteins from *P. infestans*, *P. sojae,* and *P. ramorum,* as well as the Drosha proteins from *D. melanogaster* and *H. sapiens*. Both Dcl1 and Dcl2 possess characteristic domains of the Dicer-like family (Fig 1A).

The Rdr identified in *P. capsici* contains the key domains of this protein (Fig 1B) and is most closely related to those in *P. infestans*, *P. cactorum*, *P. nicotianae*, and *P. parasitica*. The topology of the Rdr phylogeny shows that these protein sequences are more closely related among *Phytophthora* species than to Rdr6 of *A. thaliana*. Additionally, no potential *RDR*-related genes were found in species such as *P. kernoviae* or *P. palmivora*.

The maximum likelihood phylogenetic reconstruction of Exp5, using homologs from other *Phytophthora* species and the Akaike Information Criterion, indicated that Exp5 of *P. capsici* is more closely related to its homologs in *P. cactorum*, *P. infestans*, *P. nicotianae,* and *P. parasitica* (Fig 1C). Generally, the oomycete Exportin-5 clustered distantly from the homologs in *M. musculus* and *H. sapiens*. All proteins from the *Phytophthora* species contained the IBN_N, Xpo1, and Exportin-5 domains, which are crucial for their function.

**Fig 2:**
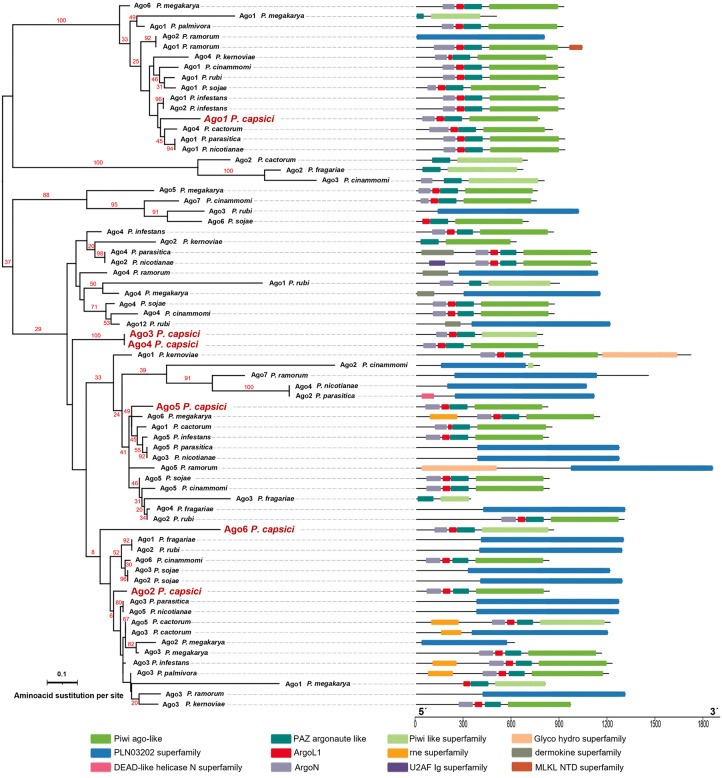
Phylogenetic reconstruction of Ago in *Phytophthora* species. Phylogenetic reconstruction of Argonaute proteins from *Phytophthora capsici* and other oomycetes. The scale bar indicates the size of the protein in amino acids. The conserved domains of the proteins are shown in color on the right side of the phylogeny.

The phylogenetic reconstruction of the six Argonautes of *P. capsici* (1, 2, 3, 4, 5, and 6) shows that these enzymes grouped into two main clades of homologs, Agos from oomycetes such as *P. infestans*, *P. sojae*, *P. cactorum*, *P. cinnamomi*, *P. fragarie*, *P. kernoviae*, *P. megakarya*, *P. nicotianae*, *P. palmivora*, *P. parasitica*, *P. rubi,* and *P. ramorum* (Fig 2).

Ago1 groups into clade I along with Ago1 sequences from *P. parasitica*, *P. megakarya*, *P. palmivora*, *P. ramorum*, *P. sojae*, and *P. infestans*. In contrast, the other Agos of *P. capsic*i fall into clade II. Ago2 mainly relates to Ago3 from *P. parasitica* and Ago5 from *P. nicotianae*. However, Ago3, 4, and 6 form a separate clade. At the same time, Ago5 shows a closer phylogenetic relationship to Ago6 from *P. megakarya*, Ago1 from *P. cactorum*, Ago5 from *P. infestans*, Ago5 from *P. parasitica*, and Ago3 from *P. nicotianae*. The primary structure of these proteins indicates that *P. capsici* argonautes and the other Ago proteins analyzed vary in size and differ in the presence of characteristic domains of argonaute proteins (ArgoN, AgoL, PAZ, and PIWI). Supporting Information 1 (S1 Table) lists the IDs and protein sequences used in the phylogenetic analyses.

The genomic neighborhood of the *P. capsici DCLs*, *AGOs*, and *RDR* genes showed that they are located on different scaffolds within gene-rich regions and some transposable element regions. However, some genes are situated relatively close to these regions (S1 Fig.). Additionally, the LocTree3v4.0 software indicates that the Dcl1 and Dcl2 proteins might be located in the nucleus and mitochondria, respectively, whereas Agos and Rdrs are likely located in the cytoplasm (Table 1).

### *P. capsici EXPORTIN-5* is located in gene-rich and conserved genomic regions

**Fig 3:**
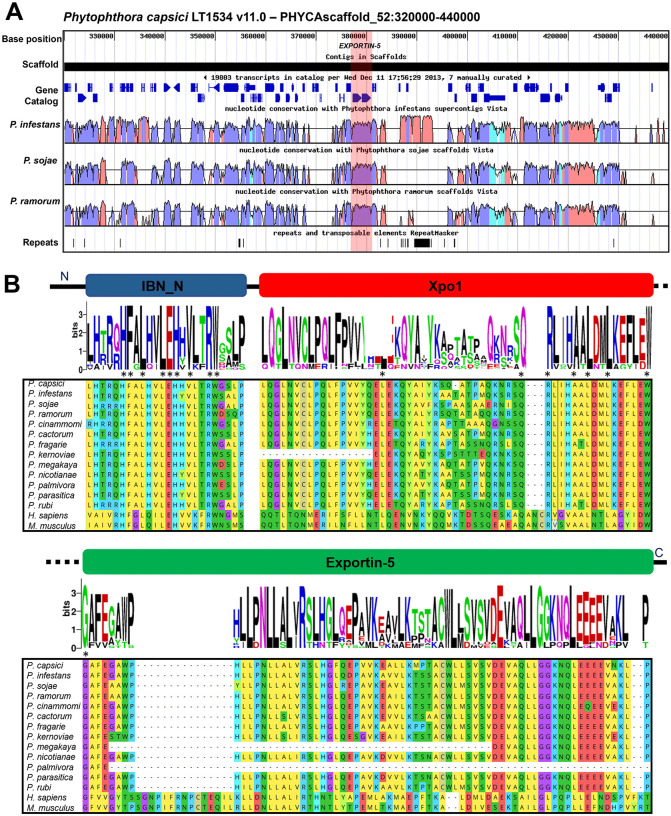
Genomic neighborhood and conserved domains of Exportin-5. **(A)** Genomic neighborhood of *P. capsici EXPORTIN-5* gene shown in red stripe, located in gene-dense region and highly conserved regions between *P. infestans*, *P. sojae,* and *P. ramorum* genomes, conserved region cyan: UTR, blue: exons, red: introns. **(B)** Sequence alignment of exportin-5 proteins divided by the conserved domains IBN_N, Xpo1, and Exportin-5 in various species of *Phytophthora*, *H. sapiens,* and *M. musculus*, linked to a weblogo plot of abundance of amino acids at a determined position. * Amino acid conservation among all ranked organisms. - Amino acid misalignment.

To determine the genomic neighborhood of the *P. capsici EXP5* gene, we used the JGI browser tool and compared it with the genomes of *P. infestans*, *P. sojae*, and *P. ramorum* (Fig 3A). We found that the gene is located on scaffold 52:377206–380861 (+) in *P. capsici* LT1534. The *EXP5* gene appears to be in a gene-rich region, with neighboring genes on the left and right corresponding to a drug/metabolite transporter (DMT) and a galactosyltransferase, respectively. *EXP5* contains two exons and is highly similar to other homologous genes in the genomes of *P. sojae* (ID 465489, Physo3) and *P. ramorum* (ID 81240, *P. ramorum* v1.1).

Global alignment of the *P. capsici* Exp5 protein with homologs from *M. musculus* and *Homo sapiens* showed 24.6% and 24.2% identity, and 41% and 41.5% similarity, respectively. However, among oomycetes such as *P. infestans*, *P. sojae*, and *P. ramorum*, the identity was 86.2%, 86.8%, and 87.2%, with 92.9%, 93%, and 92.8% similarity, respectively. Domain analysis of *P. capsici* Exp5 revealed that it contains the three characteristic domains of Exportin-5: IBN_N, Xpo1, and Exportin-5.

Multiple sequence alignment of oomycete Exportin-5 proteins and their *H. sapiens* and *M. musculus* homologs revealed decreased similarity and differences in the amino acid residue composition of the Xpo1 and Exportin-5 domains (Fig 3B). Likewise, *P. kernoviae* showed the loss of a large region of this domain. The Exportin-5 domain was also identified as the most variable between oomycetes and mammals, and *P. megakarya* and *P. palmivora* had smaller domains than those of the other organisms (Fig 3B).

### Genes of the RNAi pathway of *P. capsici* revealed synteny with four oomycete genomes and the formation of protein interaction networks

**Fig 4:**
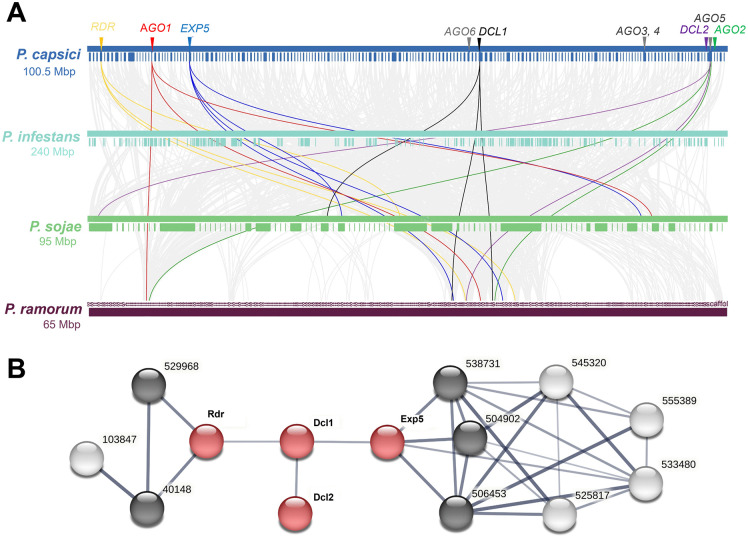
Synteny and protein-protein interaction networks of key genes in the RNAi pathway. **(A)** Synteny of key genes in the RNAi pathway in four oomycete species. Gray lines in the background indicate collinear blocks within the *P. capsici* and other oomycete genomes, whereas the colored lines highlight syntenic genes. **(B)** Protein-protein interaction networks of Dcl1, Dcl2, Exp5, and Rdr from *P. capsici*. Red nodes: interaction network of proteins related to siRNAs in *P. capsici*; black nodes: first shell of interactions; white nodes: second shell of interactions; shell number: proteome ID from the *P. capsici* v11 JGI database. A thicker dark line indicates a higher score for protein-protein interaction. IDs: 504092 (GTPase Ran/TC4/GSP1 nuclear protein transport), 506453 (trafic intracellular, nuclear pore complex, Nup214/CAN component), 538731 (Ran GTPase-activating protein, RNA processing and modification), 525817 (ubiquitin-protein ligase), 545320 (karyopherin importin beta 1, intracellular trafficking, secretion and vesicular transport), 555389 (nuclear export signal-RNA export factor), 533480 (nuclear porin, structural constituent of nuclear pore), and 103847 (DNA excision repair protein XPA/XPAC/RAD14).

The synteny analysis of the genomes of *P. capsici*, *P. infestans*, *P. sojae*, and *P. ramorum* indicated that the genes *DCL1*, *DCL2*, *EXP5*, *AGO1*, *AGO2*, and *RDR* are syntenic among all four genomes (Fig 4A and S2 Table). In contrast, the *AGO3*, *AGO4*, *AGO5*, and *AGO6* genes of *P. capsici* are not syntenic with those in the other oomycetes (*P. infestans*, *P. sojae*, and *P. ramorum*) (Fig 4A and S2 Table). Overall, it seems that the *DCLs*, *EXP5*, *AGOs*, and *RDR* genes are dispersed throughout the *P. capsici* genome, similar to *P. infestans* and *P. sojae*, whereas in *P. ramorum* these genes are located close to each other.

Predictive analysis of the protein-protein interaction networks of Dcl1, Dcl2, Exp5, and Rdr (Fig 4B) suggests that they may interact within a common regulatory pathway, with a mean confidence score of 0.4. Additionally, Exp5 potentially interacts with proteins 504092 (GTPase Ran/TC4/GSP1, nuclear protein transport), 506453 (traffic intracellular, nuclear pore complex, Nup214/CAN component), and 538731 (Ran GTPase-activating protein, RNA processing and modification) in 1st shell. There is also predictive evidence that it interacts with 525817 (ubiquitin-protein ligase), 545320 (karyopherin importin beta 1, intracellular trafficking, secretion, and vesicular transport), 555389 (nuclear export signal-RNA export factor), and 533480 (nuclear porin, structural constituent of nuclear pore) in 2nd shell. Rdr potentially interacts with proteins 529968 (E3 ubiquitin ligase) and 40148 (structure-specific endonuclease ERCC1-XPF, ERCC1 complex) in the 1st shell, and with 103847 (DNA excision repair protein XPA/XPAC/RAD14) in the 2nd shell.

### *P. capsici* showed changes in virulence and different expression patterns of RNAi pathway genes after infecting chili pepper and broccoli for two generations

**Fig 5:**
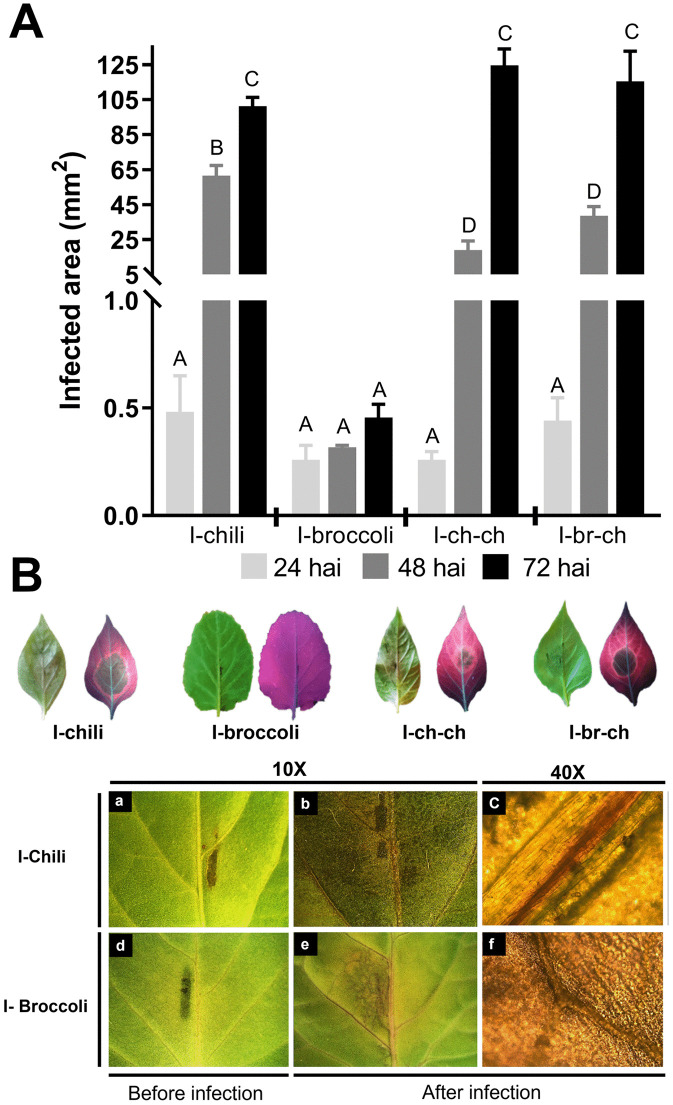
Infection caused by *P. capsici.* **(A)** Infection caused by *P. capsici* D3 in chili and broccoli leaves at 24, 48, and 72 hours post-inoculation (hpi). The bars indicate the standard error of the mean (SEM) of the infected area in mm^2^ from five independent experiments. Groups A, B, C, and D indicate ANOVA significant differences (p < 0.05) in the development of infection within each plant species. **(B)** Phenotype of *P. capsici* infection in chili and broccoli. Left leaf of each host: visualization in natural light; the dark regions show the infected area. Right leaf: visualization with UV light (340 nm); the red regions indicate healthy tissue, the dark regions show the infected area in necrosis, and the orange regions show infection development in the biotrophic phase. Infection zoom: before and after infection (72 hpi) at 10X and 40X.

To determine whether RNAi pathway genes are expressed in *P. capsici* after different infection histories over two generations, we analyzed their expression levels using RT-qPCR. First, we evaluated the development of *P. capsici* infections on chili pepper and broccoli leaves. The pathogen showed a specific infective ability on both plants, with a progressive increase in infected areas at 24, 48, and 72 hpi (Fig 5A). Chili pepper leaves were more susceptible to infection at 72 hpi. In contrast, the pathogen exhibited limited infectivity in broccoli leaves, indicating that broccoli was the most resistant plant. At 48 hpi, during secondary infections, *P. capsici* showed a statistically significant decrease in virulence (I-ch-ch and I-br-ch). However, at 72 hpi, no significant changes in virulence were observed under either condition.

Infection in chili pepper leaves was characterized by necrosis in the center and along the veins, forming dark areas, with probable biotrophy at the leaf edges; and orange coloration under UV light. However, the leaves did not show chlorosis or wilting (Fig 5B). Meanwhile, infections in broccoli leaves showed necrosis around the inoculation site, followed by a chlorotic halo, probable biotrophy around the inoculation site and before the chlorotic area, and rippling at the leaf edges (Fig 5B).

**Fig 6:**
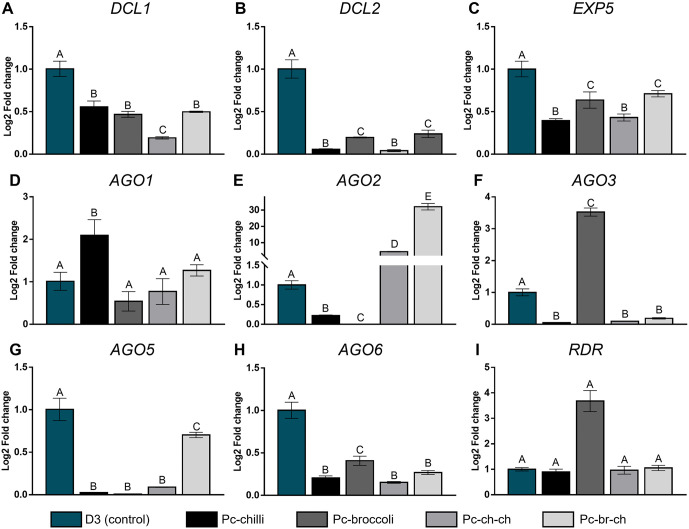
Relative expression of key genes in the RNAi pathway in *P. capsici.* **(A)** *DCL1*. **(B)** *DCL2*. **(C)** *EXP5*. **(D)** *AGO1*. **(E)** *AGO2*. **(F)** *AGO4*. **(G)** *AGO5*. **(H)** *AGO6* and **(I)** *RDR* by 2∆Ct (Log2 fold change) from the mycelium of *P. capsici* D3 (control), Pc-chili and Pc-broccoli, Pc-ch-ch and Pc-br-ch, corresponding to two biological replicates in triplicate. The bars represent the standard error. Groups A, B, C, D, and E indicate significant ANOVA differences (p < 0.05) between treatments for the same gene; EF-1α was used as an endogenous gene.

Subsequently, we identified a unique expression profile for each *P. capsici* gene across the four experimental conditions (Pc-chili, Pc-broccoli, Pc-ch-ch, and Pc-br-ch) and the control D3 (S4 Table). The highest expression of *DCL1* was observed in the *P. capsici* D3 parental strain (control), which was significantly higher than in isolates from primary (Pc-chili, Pc-broccoli) and secondary (Pc-ch-ch, Pc-br-ch) infections (Fig 6A). However, Pc-ch-ch exhibited the lowest gene expression levels among the isolates. Additionally, the expression level of *DCL2* indicated that in Pc-chili, Pc-broccoli (primary isolates), and Pc-ch-ch, Pc-br-ch (secondary isolates), gene expression was significantly reduced compared to D3 (Fig 6B). Nonetheless, Pc-broccoli and Pc-br-ch had significantly higher expression levels than Pc-chili and Pc-ch-ch, respectively.

On the other hand, the relative expression level of *EXP5* in *P. capsici* (Fig 6C) showed a pattern similar to that of *DCL2*; that is, in D3, the expression of the gene was significantly higher than in isolates from primary and secondary infections (Pc-chili, Pc-broccoli, Pc-ch-ch, and Pc-br-ch). Additionally, the expression level of *EXP5* was significantly higher in Pc-broccoli and Pc-br-ch than in Pc-chili and Pc-ch-ch, with no significant differences between Pc-broccoli and Pc-br-ch, or between Pc-chili and Pc-ch-ch. When analyzing the relative expression profile of *AGO1* (Fig 6D), it was found that Pc-chili expressed the gene significantly higher than D3, Pc-broccoli, Pc-ch-ch, and Pc-br-ch, with no significant differences among the latter.

Meanwhile, the *AGO2* expression profile (Fig 6E) varied significantly across treatments according to ANOVA analysis. Specifically, in Pc-chili and Pc-broccoli, *AGO2* expression was lower than in the control, with no gene expression detected in Pc-broccoli. In contrast, *AGO2* was expressed at significantly higher levels in Pc-ch-ch and Pc-br-ch than in D3, showing a progressive increase in expression. The expression levels of *AGO2* were up to 3.5 times higher in Pc-ch-ch and 33 times higher in Pc-br-ch than in D3. No evidence of relative *AGO3* expression was observed. Conversely, the *AGO4* expression profile (Fig 6F) showed a significant increase in relative expression in Pc-broccoli compared to D3 (control). However, Pc-chili, Pc-ch-ch, and Pc-br-ch were expressed significantly less than the control, with no significant differences between them.

Our results also showed that the relative expression level of *AGO5* was higher in D3 than in Pc-chili, Pc-broccoli, Pc-ch-ch, and Pc-br-ch (Fig 6G). In contrast, the expression levels of the latter were higher than those of Pc-chili, Pc-broccoli, and Pc-ch-ch, with no significant differences among them, whereas *AGO6* expression decreased in the four evaluated treatments (Fig 6H). However, Pc-broccoli expression was higher than that of Pc-chili, Pc-ch-ch, and Pc-br-ch, with no significant differences in *AGO6* expression in the latter. In contrast, the relative expression profile of the *RDR* gene (Fig 6I) showed similar levels of expression among the control D3, Pc-chili, Pc-ch-ch, and Pc-br-ch, but it was significantly higher in Pc-broccoli.

## Discussion

Epigenetic mechanisms based on RNAi allow precise, dynamic, and reversible control of gene expression without altering the DNA sequence. They are essential for living organisms [69] because they influence development [70], responses to biotic and abiotic stress [71], antiviral defense [72], silencing of transposable elements [73], and evolution [74].

Recently, the role of RNAi in regulating oomycete infections has gained attention [18,20,40]. Therefore, understanding the molecular basis of siRNA synthesis, transport, and processing is essential for clarifying how virulence is regulated in plant pathogens, such as *P. capsici* [13,75]. In this study, we aimed to identify the genes and protein sequences of key enzymes involved in the RNAi pathway in *P. capsici* and analyze their relative gene expression levels after infection across different plant species.

Overall, our results revealed the presence of genes encoding key enzymes involved in RNAi regulatory pathways in the *P. capsici* genome, including two dicer-like (*DCL1* and *DCL2*), one exportin-5 (*EXP5*), six argonaute (*AGO1, AGO2, AGO3, AGO4, AGO5, and AGO6*), and one RNA-dependent RNA polymerase (*RDR*) gene. These genes are conserved among oomycetes [17] and are homologs to those in phylogenetically distant organisms such as *A. thaliana*, *H. sapiens*, and *M. musculus*. These findings suggest that *P. capsici* may use canonical sRNA-mediated epigenetic regulation similar to that reported in mammals, plants, humans, insects, fungi, and other pathogenic oomycetes [76]. However, further studies are needed to confirm the presence of small RNA-based and other epigenetic mechanisms in *P. capsici*.

### Phylogenetic analysis and functional diversification of *P. capsici* siRNA regulatory enzymes

Phylogenetic analysis revealed that Dcl1 and Dcl2 protein sequences clustered into two separate groups, with Dcl1 related to the Dcl1 group of oomycetes and Dcl2 associated with Dcl2 in *P. infestans*, *P. sojae*, and *P. ramorum*. This suggests that *P. capsici* Dcl proteins may have a different evolutionary origin, as reported for Dcl1 and Dcl2 in several *Phytophthora* species [77], probably because of the functional divergence of the enzymes. For instance, the Dcl1 homolog in *P. infestans* is involved in the production of 21 nt siRNAs in the miRNA pathway [19], while the Dcl2 homologs in *P. infestans*, *P. sojae*, and *P. ramorum* probably generate 25-nt siRNAs through the RNAi pathway [19,53,78].

Conversely, phylogenetic analysis of Exportin-5 (Exp5) from *P. capsici* indicated that it belongs to a diverse oomycete-specific clade, distinct from mammalian Exportin-5 proteins. This suggests evolutionary divergence and potential functional specialization within the gene family. Notably, this is the first report of the presence and expression of a homolog of Exp5 in *P. capsici* and other oomycetes; therefore, the evolutionary origin and function of oomycete Exp5 remain unknown. However, in mammals, Exportin-5 is a protein in the caryopherin family responsible for transporting miRNAs through the nuclear pore from the nucleus to the cytoplasm in the presence of the Ran-GTP4 cofactor [76]. Recent studies on Exportin-5 in humans focus on comprehensive analyses of XPO5 binding selectivity to different pre-miRNAs and its clinical relevance in relation to ischemic heart disease and liver cancer [79,80].

Interestingly, *P. capsici* encodes six *AGO* genes, while *P. infestans*, *P. ramorum*, *P. sojae*, and *P. parasitica* encode five, six, nine, and five *AGOs*, respectively. The Agos of *P. capsici* contain domains (ArgoL, ArgoN, MID, PAZ, and PIWI) commonly found in functional Argonaute proteins [19,79]. Phylogenetic analysis grouped Ago1 from different oomycetes into clade I, whereas the other five enzymes (Ago2–Ago6) clustered into the more diverse clade II. Clade I Agos may be miRNA-specific enzymes, whereas clade II Agos may primarily process siRNAs [18,19]. Although this may also be the case for *P. capsici*, additional experiments are needed to demonstrate it. Similarly, Ago3 and Ago4 of *P. capsici* clustered into separate clades, indicating paralogous divergence. However, our expression results suggest that Ago3 may be non-functional or only conditionally regulated, as significant relative expression was observed only for Ago4.

In contrast, Ago2 and Ago5 were associated with Agos from different oomycetes, whereas Ago6 was associated with a distinct or novel clade, suggesting potential new functional roles for oomycete Agos. These phylogenetic relationships of Ago proteins suggest gene expansion and possible functional specialization in sRNA regulatory pathways in *Phytophthora* species, which calls for further investigation. Finally, regarding the Rdr proteins, our results show that *P. capsici* encodes a single *RDR* gene, as reported in other oomycetes, including *P. infestans*, *P. sojae*, and *P. ramorum* [17]. Here, we also identified this gene for the first time in *P. megakarya*, *P. cinammomi*, *P. rubi*, and *P. nicotianae*, expanding the known distribution of *RDR*s within this genus. In contrast, the phytopathogenic fungus *Verticillium dahliae* has been reported to encode three Rdrs, whereas *A. thaliana* has six Rdr homologs [19], suggesting that Rdrs may have specific functions in different organisms and distinct evolutionary origins, as well as unique regulatory pathways among living organisms. Notably, the Rdr protein in *P. capsici* is phylogenetically distant from other oomycete Rdrs, indicating sequence differences between oomycetes. However, it remains unclear whether these differences lead to functional specificity. Based on the functions reported for members of the Rdr enzyme family, it is possible that, similar to mammals, where Rdr is involved in the biogenesis of secondary siRNAs from Ago-trimmed mRNA fragments [19,22], *P. capsici* Rdr enhances gene silencing via Ago.

However, the genomes of *P. kernoviae* and *P. palmivora* do not encode *RDR* homologs. This is probably due to a combination of functional reduction, replacement by other regulatory pathways, specific evolutionary pressures, evolutionary loss due to redundancy, and lower exposure to transposons or viruses, among other factors [19]. It is possible that these genes are simply missing from current assemblies rather than being biologically absent.

Identifying these key enzymes in the RNAi pathway of *P. capsici* opens the door to further studies that analyze their functions, thereby providing a deeper understanding of their roles in the lifestyle of the pathogen.

### Synteny, genomic organization, and protein-protein interaction networks of *P. capsici* siRNA regulatory enzymes

The *P. capsici* genes encoding key enzymes of the RNAi pathway showed syntenic relationships with homologous genes in other oomycetes, and the encoded proteins may interact with other proteins within complex networks. The *DCL1, DCL2*, *EXP5*, *AGO1*, *AGO2*, and *RDR* genes of *P. capsici* exhibited collinearity and were syntenic with corresponding regions in the genomes of *P. infestans*, *P. sojae*, and *P. ramorum*, indicating conserved regulatory loci. A syntenic relationship has been reported for argonaute genes from *A. thaliana* and pineapple [81,82], as well as for some oomycete *AGO* genes [17]. However, for oomycetes, co-localization and syntenic conservation have been reported only for *AGO3*, *AGO4*, and *AGO5* in *P. infestans*, *P. sojae*, and *P. ramorum* [17]. Synteny analyses between the genomes of *P. infestans*, *P. sojae*, *P. ramorum*, and *P. betacei* have revealed regions of high synteny in core genes and high plasticity in host-pathogen interaction genes, as well as segmental duplication events [60,83]. Similarly, it has been observed that *NRL* genes of *P. infestans* are syntenic across the genomes of potato, *Arabidopsis*, tomato, and rice [84]. Therefore, our findings expand the understanding of conserved syntenic gene families involved in siRNA epigenetic regulatory mechanisms in oomycetes.

Regarding interaction networks, the results suggest that Dcl1, Dcl2, Exp5, and Rdr proteins may participate in interconnected silencing regulatory pathways. Different Dcl-Ago-Rdr combinations work synergistically to silence specific RNAs that control invading nucleic acids from either endogenous or exogenous origin, a process mediated by various siRNAs [85,86]. Interestingly, Exp5 was found to potentially interact with the Ran/TC4/GSP1 GTPase and various nuclear pore proteins, suggesting a transport mechanism similar to the yeast exportin system [87]. Overall, these data indicate a complex network of interactions among key epigenetic regulatory enzymes in *P. capsici*, which are syntenically related to those of other pathogenic oomycetes.

### Host-dependent expression of *P. capsici* sRNA-related genes

There was differential expression of siRNA-related genes in *P. capsici* isolated from infected chili pepper and broccoli leaves. Generally, chili pepper was more susceptible to the pathogen than broccoli during the first infection, showing necrotic areas around the inoculation sites. These results are consistent with previous findings showing differences in aggressiveness levels across hosts [75,88–90]. Moreover, *P. capsici* was isolated from broccoli leaves far from the inoculation site, which exhibited signs of infection, including small necrotic spots and decreased fluorescence. This indicates that, under controlled laboratory conditions, the pathogen can slowly infect broccoli leaves, a plant species often considered a non-host of *P. capsici* [91]. It has been reported that broccoli synthesizes volatile isothiocyanates and other hydrolysis products with biocidal properties that come into direct contact with pathogens, preventing the infectious agents from infecting the plant [92,93].

Our study showed that the central enzymes involved in the biogenesis, transport, and processing of siRNAs are expressed in *P. capsici*. Specifically, the analysis of *DCL*s, *AGO*s, *EXP5*, and *RDR* expression in *P. capsici* mycelium suggests that these enzymes are potentially functional in D3, Pc-chili, Pc-broccoli, Pc-ch-ch, and Pc-br-ch. However, host and infection history significantly altered the expression of these genes in both primary and secondary *P. capsici* isolates, potentially influencing gene regulation. In general, our data on the expression of *DCL1*, *DCL2*, and *EXP5* indicate less regulation of gene expression in Pc-ch-ch and, consequently, *P. capsici* may expresses more genes than Pc-br-ch. Our results suggest that when *P. capsici* is absent from infecting any plant species (D3), gene regulation mediated by *DCL1*, *DCL2*, and *EXP5* is significantly increased. This could be because *P. capsici* D3 grows optimally on culture media supplemented with nutrients from various plants. Experiments on the *DCL1* and *DCL2* genes in *P. infestans* [94] and *P. sojae* [77] showed no significant differences in relative expression levels when growing on culture media. Likewise, the two Dcl enzymes (EqDCL1 and EqDCL2) of the obligate parasitic fungus *Erysiphe quercicola* are associated with the infection process of the rubber tree (*Hevea brasiliensis*) [95]. Here, we found differences in relative expression depending on the previously infected host, supporting the idea that host and infection history in *P. capsici* influence the expression of *DCL1* and *DCL2*.

The results of the relative expression of argonaute genes (*AGO1*, *2*, *3*, *4*, and *6*) suggest that they could play important roles in *P. capsici*, as shown by significant changes in their expression between treatments and the control, suggesting that they have distinct functions. Variations in the transcript levels of different *AGO* genes have also been observed in the mycelia of *P. sojae* and *P. infestans* [77,96], further emphasizing the differences in the potential functions of *AGO* genes in oomycetes.

Meanwhile, in *P. parasitica*, there is strong evidence that *AGO3* plays a key role in virulence [18]. Therefore, we believe that the changes in the expression levels of different *AGO*s in *P. capsici* could be partly linked to the changes in pathogenicity observed during infection experiments, as potential sRNAs processed from the different *AGO*s might regulate effector genes [19], with the plant species and infection history also affecting the virulence of *P. capsici*. However, when the oomycete is recovered from infecting a first or mixed host, such as chili pepper and broccoli, it generally results in decreased gene expression compared to the control, with some exceptions. This suggests that oomycete gene expression increases due to reduced argonaute gene expression. That is, the expression of argonaute genes is influenced by the host through memory and possibly epigenetic inheritance, since at the time of gene expression analysis, the oomycete was not directly interacting with the host. Overall, the data on argonaute gene expression in *P. capsici* are of great interest, as their role in oomycete pathogenicity was previously unknown. It is now known that the pathogen exhibits host-induced genotypic plasticity, driven by changes in argonaute gene expression.

Finally, we observed variation in *RDR* expression across the studied treatments, indicating that broccoli infection significantly increased *RDR* expression compared to the control D3, Pc-chili, Pc-ch-ch, and Pc-br-ch. The importance of *RDR* expression in the vegetative mycelium of *P. sojae* has also been examined [77], revealing distinct sRNA-mediated silencing pathways within the genus *Phytophthora*. These relative expression experiments are highly relevant, as it was previously unknown whether *P. capsici* expressed the key enzymes involved in the RNAi regulatory pathway. The next step is to perform functional experiments on these enzymes.

### Hypothetical model of *P. capsici* RNAi pathway

**Fig 7:**
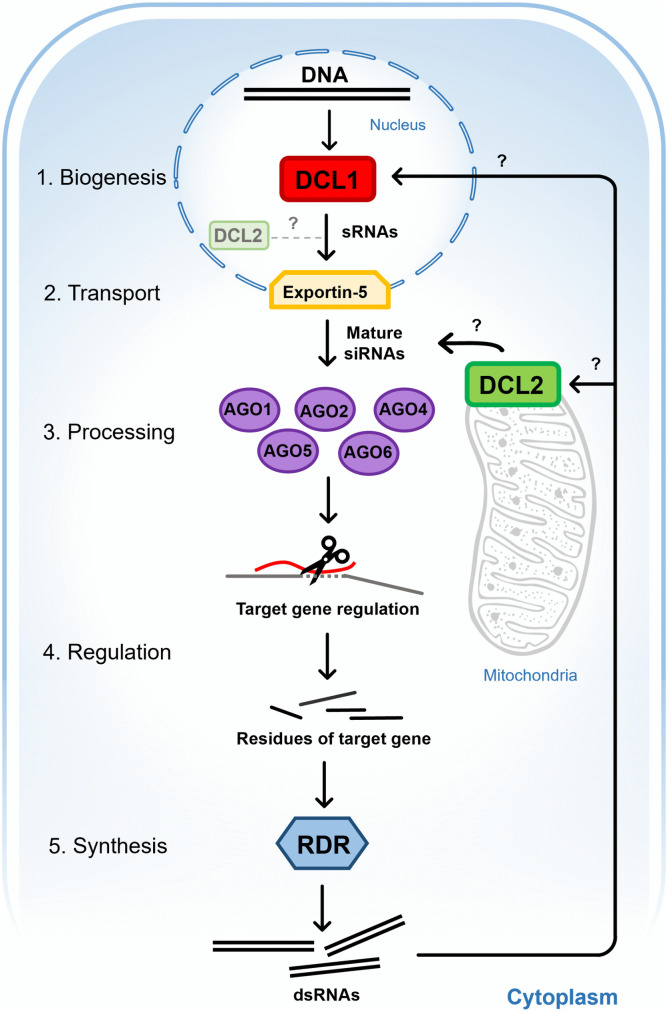
Hypothetical model of biogenesis, transport, and processing of small interference RNAs in *P. capsici.* 1. Biogenesis of small interference RNAs by Dcl1 and Dcl2, 2. Transport of small interference RNAs by Exp5, 3. Processing of mature siRNAs, 4. Regulation of target genes by Agos, 5. Synthesis of dsRNAs by Rdr to amplify the gene silencing signal.

Our predictive results indicate that Dcl1 is located in the nucleus and Dcl2 in the mitochondria, which contrast with the findings in *P. infestans* and *P. sojae*, where both Dcl1 and Dcl2 were found in the nucleus of these oomycetes [53,97]. Then, the localization of Dcl2 in *P. capsici* could be in both the nucleus and mitochondria, since our results on mitochondrial localization are based solely on *in silico* predictions, and further *in vivo* studies are required to confirm its subcellular localization. Furthermore, there is a possibility that Dcl2 of *P. capsici* is associated with mitochondria, since reports of mitoRNAs [98,99] suggest that Dicer enzymes might be associated with the mitochondrial membrane. Therefore, further research is needed to experimentally validate the subcellular localization of Dcl1 and Dcl2 in *P. capsici*. Exp5 was identified as being located in the nucleus, and the Agos in the cytoplasm. Based on data on the predictive subcellular localization, expression profile, and related literature on siRNA biogenesis and processing enzymes in oomycetes [17,18,96,100], we constructed a hypothetical model of the biogenesis, transport, and processing of siRNAs in *P. capsici* influenced by Dcls, Exp5, Agos, and Rdr (Fig 7). This model includes five main steps: 1) Potential biogenesis of small interference RNAs in the nucleus and mitochondria by Dcl1 and Dcl2, respectively, from transcribed DNA; 2) siRNAs could be transported to the cytoplasm by Exportin-5 and potentially recognized and loaded by one of the argonaute enzymes (Ago1, 2, 4, 5, 6); 3) Processing of mature siRNAs; 4) Regulation of target genes; and 5) RNA-dependent RNA polymerase (Rdr) synthesizes dsRNA from target gene residues and transports them via an unknown system to Dcl enzymes to amplify the regulatory signal of a specific target gene.

## Conclusion

Our study provides the first comprehensive identification and expression profiling of core components of the RNA interference pathway in *P. capsici*. The *P. capsici* genome encodes essential enzymes involved in RNAi, including two *DCL*s, one *EXP5* gene, six *AGO*s, and one *RDR* gene, all phylogenetically related to those in other oomycetes. Some of these genes are syntenic with homologous genes from other oomycetes. The presence and expression of these genes, especially *EXP5* and *RDR*, which have not been previously reported in this species, highlight their potential roles in gene regulation during infection. Notably, we observed host-specific changes in gene expression, which may reflect transcriptional plasticity that contributes to the virulence and adaptation of *P. capsici* to both natural and non-host plants. However, the mechanisms underlying RNAi epigenetic regulation by these genes are complex and require further investigation, particularly to confirm functional diversification across different gene families. Finally, the present research expands knowledge of RNAi-mediated gene regulation in *P. capsici*, paving the way for new strategies to control this pathogen and providing a foundation for future functional studies on the RNA silencing machinery in oomycetes.

## Supporting information

S1 Table Protein IDs and sequences used for phylogenetic reconstruction. Protein sequences and IDs used for phylogenetic reconstruction of Dcl, Exportin-5, Rdr and Ago.
(XLSX)

S1 Fig Genomic localization of key genes in pathway of small RNAs. Genomic localization of argonaute, dicer-like, exportin-5, and RNA-dependent RNA polymerase genes in *Phytophthora capsici.* Black lines indicate genes, blue: repeats.
(TIF)

S2 Table Locus synteny of key genes in pathway of small RNAs. Locus synteny of *DCL1*, *DCL2*, *EXP5*, *AGO1*, *AGO2*, *AGO3*, *AGO4*, *AGO5*, *AGO6* and *RDR* of *P. capsici* versus *P. infestans, P. sojae* and *P. ramorum genomes.* X indicate No synteny.
(XLSX)

S3 Table Primers for RT-qPCR. Characteristics of primers and experimental conditions used to perform RT-qPCR of genes *DCL1*, *DCL2*, *EXP5*, *AGO1*, *AGO2*, *AGO3*, *AGO4*, *AGO5*, *AGO6*, *RDR* and *EF-1α* in *P. capsici*.
(XLSX)

S4 Table Ct value. Ct value of *DCL1*, *DCL2*, *EXP5*, *AGO1*, *AGO2*, *AGO3*, *AGO4*, *AGO5*, *RDR* and *EF-1α* genes of *P. capsici* in RT-qPCR.
(XLSX)
